# DNN-Based Assistant in Laparoscopic Computer-Aided Palpation

**DOI:** 10.3389/frobt.2018.00071

**Published:** 2018-06-19

**Authors:** Tomohiro Fukuda, Yoshihiro Tanaka, Michitaka Fujiwara, Akihito Sano

**Affiliations:** ^1^Department of Electrical and Mechanical Engineering, Graduate School of Engineering, Nagoya Institute of Technology, Nagoya, Japan; ^2^Japan Society for the Promotion of Science, Tokyo, Japan; ^3^Department of Gastroenterological Surgery, Graduate School of Medicine, Nagoya University, Nagoya, Japan

**Keywords:** detection assistance, tumor, tactile sensor, deep neural network, laparoscopy

## Abstract

Tactile sensory input of surgeons is severely limited in minimally invasive surgery, therefore manual palpation cannot be performed for intraoperative tumor detection. Computer-aided palpation, in which tactile information is acquired by devices and relayed to the surgeon, is one solution for overcoming this limitation. An important design factor is the method by which the acquired information is fed back to the surgeon. However, currently there is no systematic method for achieving this aim, and it is possible that a badly implemented feedback mechanism could adversely affect the performance of the surgeon. In this study, we propose an assistance algorithm for intraoperative tumor detection in laparoscopic surgery. Our scenario is that the surgeon manipulates a sensor probe, makes a decision based on the feedback provided from the sensor, while simultaneously, the algorithm analyzes the time series of the sensor output. Thus, the algorithm assists the surgeon in making decisions by providing independent detection results. A deep neural network model with three hidden layers was used to analyze the sensor output. We propose methods to input the time series of the sensor output to the model for real-time analysis, and to determine the criterion for detection by the model. This study aims to validate the feasibility of the algorithm by using data acquired in our previous psychophysical experiment. There, novice participants were asked to detect a phantom of an early-stage gastric tumor through visual feedback from the tactile sensor. In addition to the analysis of the accuracy, signal detection theory was employed to assess the potential detection performance of the model. The detection performance was compared with that of human participants. We conducted two types of validation, and found that the detection performance of the model was not significantly different from that of the human participants if the data from a known user was included in the model construction. The result supports the feasibility of the proposed algorithm for detection assistance in computer-aided palpation.

## Introduction

Surgical skills involve manipulation and palpation. Manipulations such as a needle insertion, making incisions, or suturing a wound, are based on the surgeon's sensory-motor control for achieving the desired movements of surgical instruments, or handling of target tissue. Palpation is also based on sensory-motor control, but ultimately it is aimed at obtaining information about the target tissue, or detecting hard masses such as tumors in soft tissue. Thus, these skills rely on sensory inputs from visual, auditory, and tactile channels (Okamura et al., 2011), and a surgeon efficiently integrates these sensory inputs. However, in certain medical fields there are situations where the sensory input is restricted. For example, minimally invasive surgery such as laparoscopic surgery substantially limits the tactile sensory input of the surgeon, therefore there are difficulties in dexterous manipulation and palpation.

Computer-aided surgery is one solution to overcome these difficulties owing to the lack of tactile feedback. Robotic technologies can assist and enhance the manipulation ability of a surgeon, by facilitating high precision and multiple degrees of freedom within a small working area. Moreover, autonomous surgery by robots has attracted attention. Surgical robots with active constraints (Bowyer et al., 2014), and robots for autonomous suturing (Pedram et al., 2017), are both examples of achieving a high level of autonomy. Some research studies have also tried to achieve a high level of autonomy in computer-aided palpation. For instance, Hui and Kuchenbecker (2014) evaluated the ability of BioTac® for the detection and characterization of lumps in soft objects, as a step toward the development of an automatic palpation tool. McKinley et al. (2015) developed a disposable palpation probe that is mounted on the tip of a da Vinci® tool, and Garg et al. (2016) achieved autonomous tumor localization by using this probe. Konstantinova et al. (2017) achieved autonomous tumor detection with robotic manipulation of a sensor probe, on the basis of human palpation strategies. However, it was only successful under controlled conditions such as using known target tissues, and autonomous palpation is still challenging owing to the complex and variable sensing environments. For practical applications, direct control of the sensing device by a surgeon is preferable, to ensure the safety of the patient. Moreover, ethical and legal issues will be significant hurdles to overcome before widespread application is achieved. Non-autonomous computer-aided palpations are still advantageous, because they can be considered as natural extensions of current surgical procedures. In these palpations, the surgeons make the decision based on information given by the systems and the operation. Thus, the method by which the acquired information is fed back to a surgeon is an important design factor (Culmer et al., 2012).

There are many research works on non-autonomous computer-aided palpation. For instance, there are hand-held devices that are directly manipulated by a surgeon to acquire information about the target tissue (Ottermo et al., 2004; Schostek et al., 2006; Beccani et al., 2014; Escoto et al., 2015; Solodova et al., 2016). Moreover, master–slave surgical systems with a force/tactile sensor (Tavakoli et al., 2006; Talasaz and Patel, 2013; Meli et al., 2016; Pacchierotti et al., 2016; Li et al., 2017) or force estimation by a state observer (Gwilliam et al., 2009; Yamamoto et al., 2012; Schorr et al., 2015) have been developed. In addition, a training simulator for femoral palpation and needle insertion was developed (Coles et al., 2011). Among the above-mentioned systems it is common to provide visual feedback, such as by displaying a color map (Schostek et al., 2006; Talasaz and Patel, 2013; Beccani et al., 2014; Escoto et al., 2015; Solodova et al., 2016; Li et al., 2017), a graphical bar (Gwilliam et al., 2009; Schorr et al., 2015), a sequential lamp (Tavakoli et al., 2006), or a color map overlaid on an endoscopic image (Yamamoto et al., 2012). As an additional approach for sensory feedback, a tactile display (Ottermo et al., 2004; Coles et al., 2011; Schorr et al., 2015; Pacchierotti et al., 2016), or force feedback through a master console (Tavakoli et al., 2006; Gwilliam et al., 2009; Schorr et al., 2015; Meli et al., 2016) have either been developed or implemented.

We have focused on temporal information-based palpation, in which time series of outputs from a tactile sensor are fed back to the user, to enable intraoperative tumor detection in laparoscopic surgery. A major advantage of temporal information-based palpation systems is that they can be simple, as they use of a single sensing element. For our system, we developed a forceps-type tactile sensor using an acoustic sensing principle (Tanaka et al., 2015). This sensor has important advantages for surgical applications: there are no electrical elements within the portion inserted into the patient's body, it can be sterilized, and it is disposable. Moreover, we investigated the effects of visual and tactile feedback from the sensor, which was being directly manipulated by the user for laparoscopic tumor detection (Fukuda et al., 2018). A line graph showing the time series of the sensor output was provided as the visual feedback. We have developed a tactile display that presents a force to the upper side of the foot of the user to provide tactile feedback. The foot was chosen because it is an unclean area of the surgeon, therefore the display does not need sterilization. It was found that the visual feedback significantly enhanced tumor detection. The tactile feedback had a positive effect of reducing the scanning speed during detection, but it did not significantly enhance the detection sensitivity. We speculate that after further improvement, tactile feedback will be an effective tool for tumor detection. Currently, visual feedback is an informative approach for detection, because users can discriminate a tumor based on the shape of the line graph. However, visual feedback can be problematic, as it requires an extra monitor which would occupy valuable space in the operating room. Further, a major concern is the possibility of visual sensory overload (Richard and Coiffet, 1995), because primarily the surgeon must concentrate on the laparoscopic image.

In this study, we propose an assistance algorithm for computer-aided palpation. Our scenario is that the surgeon manipulates a sensor probe and makes a decision based on the temporal sensory feedback given by a tactile display, and simultaneously the assistance algorithm performs detection based on the time series of the sensor output. Thus, the algorithm assists in the decision making process of the surgeon, by providing independent detection results. This method is advantageous because the reliability and safety of the manipulation are ensured by a human operator, but a more effective detection might be expected from the collaboration between a human operator and the algorithm. In our temporal information-based palpation, if the detection performance of the algorithm could achieve a level comparable with that of the human operator, then it would imply that visual feedback could be replaced by the algorithm. This would avoid any possible sensory overload. Moreover, decision support systems, where clinical information analyzed by a computer is provided to the surgeon, can reduce clinical error and improve patient outcomes (Jia et al., 2014). Thus, our proposed algorithm has the advantages of enhancing detection performance, improving the confidence of the surgeon, and removing visual feedback.

To achieve the proposed algorithm, we focused on deep neural network (DNN) techniques. In recent years, DNNs have achieved remarkable results in medical image analyses. For example, annotation (Shin et al., 2016), segmentation (Dou et al., 2016; Kleesiek et al., 2016; Pereira et al., 2016; Setio et al., 2016; Havaei et al., 2017), and diagnosis (Shen et al., 2015; Suk et al., 2015; Cheng et al., 2016) of medical images were developed by using DNNs. These studies aimed to assist in diagnosis or planning before a surgical procedure, based on preoperatively acquired medical images. Through using DNN, some of these studies detected changes in the mechanical properties of tissue, such as cerebral microbleeds, brain tumors, breast lesions, lung nodules, and pulmonary nodules. Moreover, analysis techniques have been developed where laparoscopic images are used to classify surgical events (Varytimidis et al., 2016; Pestscharing and Schoffmann, 2017), and track (Wang et al., 2017) or classify (Zhao et al., 2017) surgical instruments. These studies targeted postoperative objectives, such as assisting in medical training, analyses, and compiling databases of recorded endoscopic images. These studies exhibit the effectiveness of applying DNN techniques to information from a visual channel. However, there are fewer studies that intraoperatively analyze temporal information from a tactile sensor for laparoscopic tumor detection. For intraoperative analysis, a method for inputting the time series of the sensor output into a DNN model should be developed, to maintain both sufficient performance of the model outcome, and adequate refresh rates.

We propose to use a DNN model with three hidden layers to segment whether the sensor output at each sampling included the information on the tumor and a method for inputting the sensor output to the DNN model considering real-time analysis. Moreover, the method of determining the detection criterion is proposed, by investigating the relationship between accuracy and various detection criteria. In this paper, we aim to investigate the feasibility of the proposed algorithm by using the data acquired in our previous psychophysical experiment (Fukuda et al., 2018). In this experiment, 12 novice participants were asked to detect a phantom of an early-stage gastric tumor, under various conditions of sensory feedback. We used the obtained sensor outputs, which were not analyzed in the previous study, as a dataset. In addition to the accuracy, the potential detection performance of the model was analyzed by employing signal detection theory. Then, we conducted two types of cross-validation: within-participant and across-participant validation. The accuracy and potential detection sensitivity of the DNN model were compared with the performance of the participants, which was analyzed in our previous study.

## Data preparation

In our previous study, we conducted a psychophysical experiment to investigate the effect of using sensory feedback on tumor detection performance and manipulation behavior (Fukuda et al., 2018). Twelve participants without any medical background participated in the experiment, and they gave their written informed consent before participation. The experimental procedure was conducted in accordance with the ethical standards of the Helsinki Declaration, and approved by the Ethical Committee of Nagoya Institute of Technology. The participants were asked to discriminate a phantom of the stomach wall with/without a tumor, by scanning with a sensor probe and receiving sensory feedback from the sensor in a simulated laparoscopic environment. Four conditions for the method of feedback were set: no feedback, visual feedback, tactile feedback, and a combination of visual and tactile feedback. Under the no feedback condition, the participants did not receive any feedback from the sensor. Under the visual feedback condition, a line graph of the sensor output on a monitor was provided to the participants. Under the tactile feedback condition, our developed tactile display provided a force against the upper side of the participant's foot according to the sensor output. Under the combination condition, the participants received both visual and tactile feedback simultaneously. It was shown that the visual feedback was significantly effective in the tumor detection. The details were provided in our previous paper (Fukuda et al., 2018). In this study, we used the data acquired under the condition of visual feedback, because we aimed to replace the participants' decision through visual channel with the algorithm. The following paragraph presents how the sensor output was collected under the visual feedback condition.

**Figure 1:**
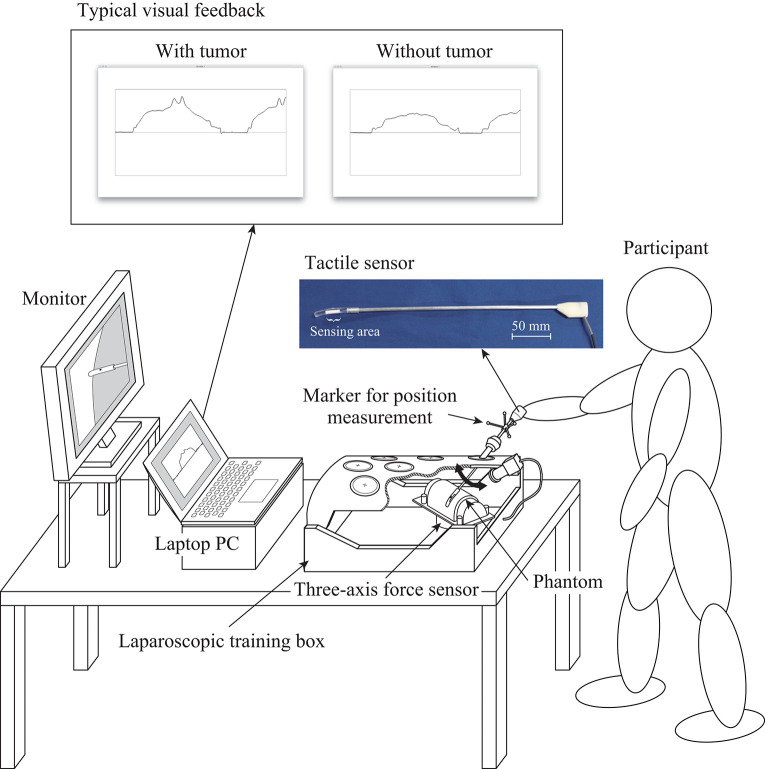
Setup of the psychophysical experiment conducted in our previous study (adapted from Fukuda et al., 2018). Participants scanned the phantom by the forceps-type tactile sensor in a rotational direction, (shown by a black bold arrow), and determined the presence of a tumor in the phantom. The rectangle shows two typical line graphs provided as visual feedback when a participant scanned a phantom with/without the tumor.

The experimental setup is shown in Figure 1. The participants manipulated a long, thin tactile sensor that was previously developed (Tanaka et al., 2015). The sensor only detected a single force applied on the side of the sensor tip. The sensor output was filtered by a low-pass filter with a 10 Hz cut off frequency. This data was then presented on a laptop PC as a line graph, for a time series of 5 s. The laptop PC was placed next to a monitor that displayed the camera image inside the laparoscopic training box. The participants scanned a phantom of the stomach wall, using the sensor in a rotational direction. There were no restrictions on either the force exerted to the phantom or the scanning speed of the sensor. The rectangle in Figure 1 shows the typical visual feedback when a participant appropriately scanned the phantom with/without a tumor. The participants could distinguish the presence or absence of a tumor based on the shape of the line graph, when they appropriately scanned the phantom. The difference in the sensor output for the two types of the phantom can also be found in the video attached as supplementary material. Before the detection trials, the participants were asked to practice the manipulation of the sensor probe, and to memorize the differences in visual feedback when scanning the phantom with/without the tumor. Then, the participants conducted 40 detection tasks comprising 20 of each scenario (presence/absence of the tumor) which were randomly generated. The force applied on the phantom was measured by a three-axis force sensor placed under the phantom, and the position and orientation of the sensor were measured by a motion capture system using a maker set attached to the sensor. The sensor output and the applied force were recorded at sampling frequencies of 1 kHz, and sensor movements were recorded at 120 Hz.

**Figure 2:**
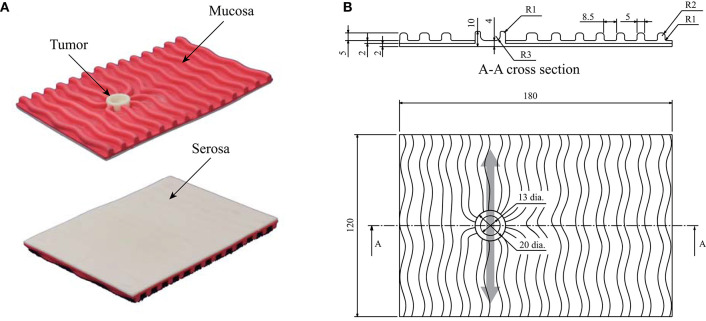
Phantom of the stomach wall used in the previous psychophysical experiment. The phantom was placed on a semi-cylindrical sponge as shown in Figure 1. **(A)** Photographs of the phantom. The serosal side (smooth side) was scanned by the sensor. **(B)** Structure of the phantom. The light gray arrow indicates that the back was scanned in the direction indicated. Thus, the typical sensor output had two small peaks, owing to the toroidal shape of the tumor.

Figure 2 shows the details of the phantom stomach wall with a tumor used in the experiment. The target tumor was a 0-IIc (superficial ulcerative) type tumor (Japanese Gastric Cancer Association, 2011), which is the most common type of early-stage gastric cancer. The geometry of the phantom and the tumor were based on the typical features of the actual stomach wall and tumor. Whereas the stiffness of the phantom was within the stiffness range of the actual stomach wall and tumor (Fukuda et al., 2018), the anatomical structures and boundary conditions were not completely the same. The tumor has a toroidal shape, therefore the sensor output typically responded with two peaks that corresponded to the two edges of the toroidal tumor, as shown in Figure 1. The phantom was placed on a semi-cylindrical sponge to simulate a stomach that does not have flat surfaces.

## Methods

### Data extraction

**Figure 3:**
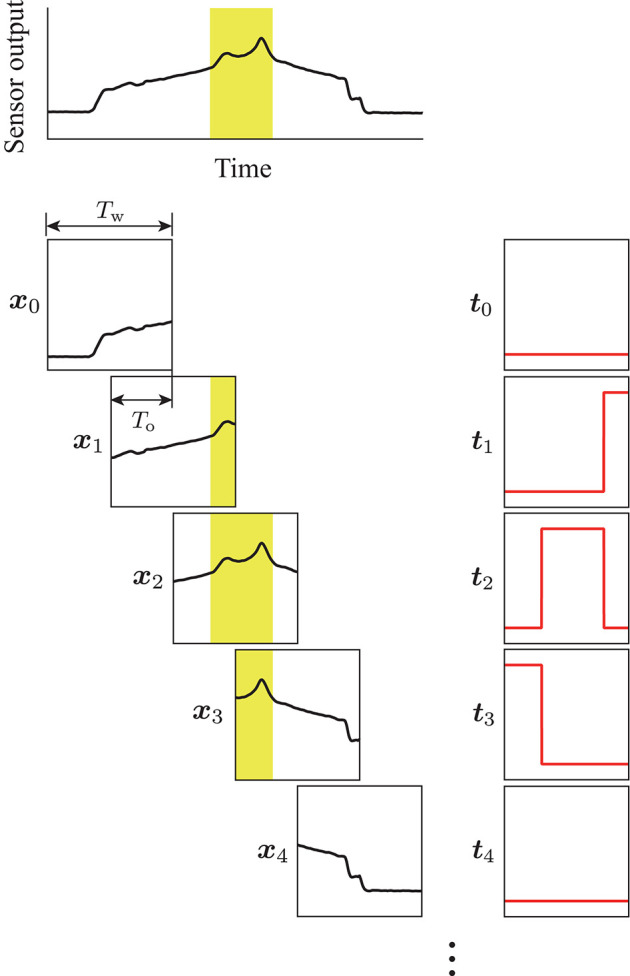
Procedure for the data extraction. Yellow areas show the tumor label, which was calculated on the basis of the sensor position measured by the motion capture system. The region of interest with a time width of *T*_w_ was shifted with the overlap time of *T*_o_. The terms ***x***_*k*_ and ***t***_*k*_ are the extracted sensor output and corresponding label, respectively. Pairs of (***x***_*k*_, ***t***_*k*_) were used for training the DNN model.

A method to input the temporal sensor output to a DNN model is important for achieving real-time analysis. For example, the use of the sampled sensor output individually is not sufficiently informative to facilitate tumor detection. Therefore, a time series is required to compare the relative differences in sensor output. However, waiting to obtain a certain length of sensor output signals leads to a reduction in the refresh rate. In this study, we propose using a region of interest to extract the sensor output, and then shift the region so that it overlaps with the previous extraction. Thus, more efficient detection can be expected by the model, while maintaining a sufficient refresh rate for detection. Figure 3 displays the procedure for data extraction. The top panel in Figure 3 shows an example of the sensor output acquired during the detection experiment. The sensor output was extracted for a time width of *T*_w_ = 1.0 s, then the extraction area was shifted so that it overlapped the previous extraction for *T*_o_ = 0.9 s. Thus, the refresh rate of the estimation achieved by the model was 10 Hz, because the data was prepared every 0.1 s and the sensor output at each sampling was included in the ten independent extractions.

For training of the DNN model, a binary label was prepared for the sensor output at each sampling to identify the sensor located above the tumor. It was calculated on the basis of both the measured position, and the orientation of the sensor during the experiment. The label was “1” while the center of the sensing area was in the tumor area, and “0” while in the other areas. The binary label was also extracted in the same way as for the sensor output. Thus, the extracted output ***x*** and the corresponding label ***t*** had the same dimension. Moreover, the time for each scanning was extracted according to the tangential force applied on the phantom tumor during the experiment. The scanning times were used in the validation of the proposed DNN model.

### DNN model

We used a deep neural network with three hidden layers. Input vector ***x*∈** ℝ^1000^ is the extracted sensor output. Output vector ***y*∈** ℝ^1000^ is the estimated tumor label corresponding to the sensor output at each sampling. The values at each hidden layer ***h***_*i*_ (***h***_0_, ***h***_4_ ∊ ℝ^1000^ and ***h***_1_, ***h***_2_, ***h***_3_ ∊ ℝ^2000^) are shown by the following equation:

$$h_{i}=\sigma \left(\;W_{i}h_{i-1}+b_{i}\right),\;(i=1,\;2,\;3,\;4),$$

where ***W***_1_ ∊ ℝ^1000×2000^, ***W***_2_, ***W***_3_ ∊ ℝ^2000×2000^, and ***W***_4_ ∊ ℝ^2000×1000^ are the weight matrices, and ***b***_1_, **b**_4_ ∊ ℝ^1000^, and ***b***_2_, ***b***_3_ ∊ ℝ^2000^ are the bias vectors. ***h***_0_ is equal to ***x***. σ indicates an element-wise activating function (rectified linear unit Nair and Hinton, 2010) as follows:

σ(z)=max(z, 0).

The output vector ***y*** was derived by applying an element-wise sigmoid function to ***h***_4_ as follows:

$$\begin{array}{l} y=\frac{1}{1+\exp\left(h_{4}\right)}. \end{array}$$

The sigmoid function was inserted to transform the value from the model to be within 0 and 1; thus, the value of each component in the output vector can be interpreted as a probability that the sensor output at corresponding sampling includes information on the tumor.

### Learning

Dataset (***x***, ***t***), which is the pair of sensor output and correct label, was used to optimize the model parameters (***W***_*i*_ and ***b***_*i*_). We used cross-entropy *E* between the correct and estimated labels for the optimization as follows:

$$\begin{array}{l} E=-\overset{\;}{\underset{k}{\sum }}t_{k}\ln\;y_{k}. \end{array}$$

where *t*_*k*_ and *y*_*k*_ are the *k*-th element of the correct and estimated labels, respectively. For the initialization of the parameters, the method proposed by He et al. (2015) was employed. The parameters were updated to minimize the cross-entropy based on the Adam method (Kingma and Ba, 2015) with a mini-batch size of 100. The update was conducted for 200 epochs.

### Detection algorithm

As described in section Data Extraction, the sensor output was extracted by a window of 1.0 s, and the window was shifted with an overlap time of 0.9 s. Thus, the proposed algorithm could provide the estimation score every 0.1 s. On the other hand, we validated the proposed algorithm by using a pre-acquired dataset; thus, we added a procedure to calculate a representative score for each experimental trial, because the participants scanned the phantom multiple times in each trial, and the number of scannings varied between participants.

**Figure 4:**
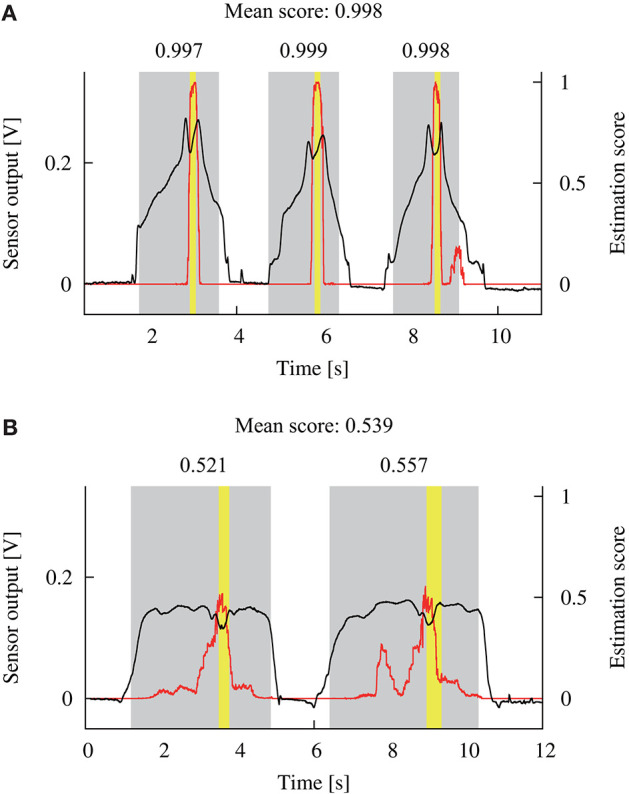
Examples of the sensor output (black lines) during a single detection trial and the estimated score (red lines) by the DNN model. Yellow areas show the time when the sensor scanned the tumor. Gray areas show the scanning time that was calculated based on the applied tangential force. The maximum estimation score within each scanning time was extracted as shown in the upper side of the gray areas. Next, the mean of the scores was calculated and used as a representative score for this trial. **(A,B)** Show the data from participants 9 and 4, respectively.

Figure 4 illustrates two examples of the sensor output and the estimation score for a single detection trial. The mean of the ten independent outputs from the DNN model was calculated for each sampling of the sensor output, as shown by the red solid lines in Figure 4. The yellow areas depict the binary label, calculated from the measured positions of the sensor. The gray areas show the extracted scanning times, based on the tangential force applied to the phantom. The maximum estimation score within each scanning was calculated as shown in the upper portion of Figure 4. Then, the mean of the maximum scores for all the scanning was calculated, and used as a representative estimation score for each trial. If the representative score was larger/smaller than the detection criterion, it was considered that the DNN model did/did not detect the tumor, respectively. This procedure allowed us to consider that the DNN model outputted a single score for each trial in a similar way to the previous experiment, where the participant's response was recorded for each trial.

**Figure 5:**
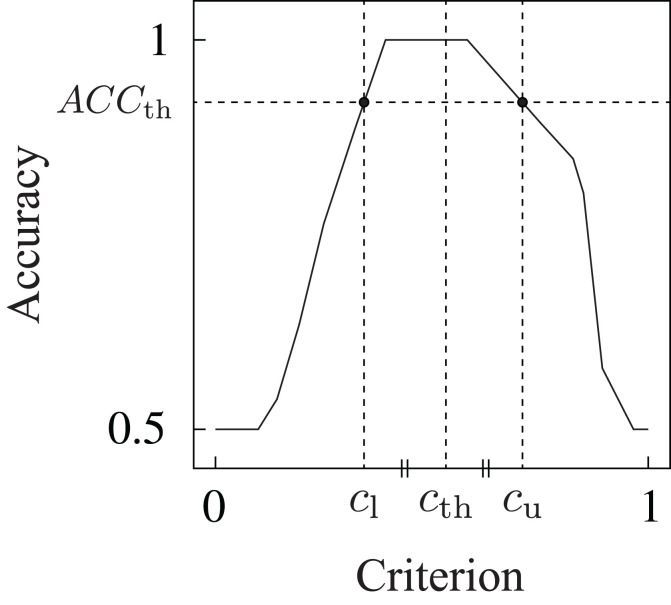
Example of the relationship between the detection criterion and accuracy. Detection criterion *c*_th_ was determined on the basis of this relationship.

We used accuracy *ACC* **∈** [0, 1] (the ratio of the number of correct detections to the number of total detection trials) as an index of the detection performance. For the determination of the detection criterion, we first investigated the relationship between the accuracy and criterion for each trained DNN model. Multiple accuracies *ACC*_*j*_ were calculated based on the estimated scores for multiple detection criteria *c*_*j*_ from 0 to 1, with 0.01 increments (*j* = 0, …, 100). The relationship between the accuracy and detection criterion was derived as shown in Figure 5. Subsequently, the threshold detection criterion *c*_th_ was calculated on the basis of the following equations:

$$\begin{array}{l} ACC_{\text{th}}=\max\left(ACC_{j}\right)-\frac{\max\left(ACC_{j}\right)-0.5}{10},\\ \;c_{\text{th}}=\frac{c_{\text{u}}(ACC_{\text{th}})+c_{\text{l}}(ACC_{\text{th}})}{2}, \end{array}$$

where *ACC*_th_ is the threshold of the accuracy to obtain upper criterion *c*_u_(*ACC*_th_) and lower criterion *c*_l_(*ACC*_th_), as shown in Figure 5. Threshold criterion *c*_th_ was calculated as the mean of the upper and lower criteria. The equation to calculate *ACC*_th_ was experimentally determined. The threshold detection criterion determined by this method leads to the maximum accuracy for the dataset used in the model construction.

### Potential sensitivity

When investigating the detection outcomes of the proposed DNN model, it is important to evaluate the accuracy. However, accuracy does not explicitly reveal how a classifier has the potential to distinguish between two situations, because the detection criterion also affects the accuracy. Thus, we additionally analyzed the detection results based on signal detection theory (Macmillan and Creelman, 2005) to assess the potential detection performance. Hit rate *H* **∈** [0, 1] (the number of detections divided by the total number of trials in which the tumor was present) and false alarm rate *F* **∈** [0, 1] (the number of detections divided by the total number of trials in which the tumor was absent) were calculated for multiple detection criteria *c*_*j*_. Hence, multiple pairs of false alarm and hit rates (*F*_*j*_, *H*_*j*_), (*j* = 0, …, 100) were derived. Then, a receiver operating characteristic (ROC) curve was drawn by plotting and connecting all the pairs of the false alarm and hit rates for the different detection criteria on the (*F, H*) space. The area under the ROC curve *A*_g_ was calculated as follows:

$$\begin{array}{l} A_{\text{g}}=\frac{1}{2}\underset{j}{\sum }\left(F_{j+1}-F_{j}\right)\left(H_{j+1}-H_{j}\right). \end{array}$$

Here, *A*_g_ indicates the potential sensitivity of a classifier, *A*_g_ lies within [0, 1], and the chance level is 0.5.

### Validation

Two types of validation were conducted on the basis of a dataset used for model training. One is within-participant validation, where the data obtained from one participant is used for both training and testing the model. The other is across-participant validation, in which to perform the detection test for the data from a single participant, the data from all other 11 participants were used in the model construction. The details of the data preparation for each validation are presented in the following sections.

#### Within-participant validation

**Figure 6:**
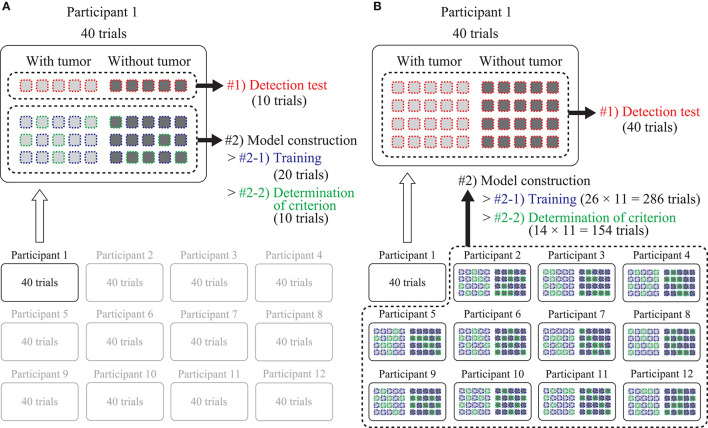
Preparation of the dataset for the two types of validations. Light and dark gray filled rectangles indicate the data acquired at a single detection trial where the tumor was present and absent, respectively. Red, blue, and green dashed lines indicate that the data was assigned for detection test, model training, and determination of the detection criterion, respectively. **(A)** Within-participant validation. Four-fold cross-validation was conducted within the data for each participant. **(B)** Across-participant validation. Leave-one-out cross-validation was conducted against data for all the participants. Both validations were conducted independently, 12 times along all the participants.

A 4-fold cross-validation was performed within the data for each participant. Figure 6A shows the procedure for the preparation of the data used for the within-participant validation. The data from one participant was divided into four groups, and each group contained the data for ten trials. The data in one group was used for the detection test (dataset #1). The data in the remaining three groups were used for the model construction (dataset #2). Moreover, dataset #2 was divided into two groups: one was for the training of the DNN model (dataset #2-1) that included 20 trials from ten of each type of trial in which the phantom with/without the tumor was presented, and the other was for the determination of the detection criterion (dataset #2-2) including ten trials from five of each. The division of dataset #2 was randomly conducted five times, regardless of the first grouping. The within-participant validation was independently conducted for 12 participants.

First, the DNN model was trained by using a pair of (***x***, ***t***) in dataset #2-1. Then, estimated label ***y*** was obtained against sensor output ***x*** in dataset #2-2 by the trained model. The estimation score was calculated from estimated label ***y*** for each trial in dataset #2-2. Accuracies *ACC*_*j*_ for the multiple detection criteria were calculated, and the threshold detection criterion *c*_th_ was determined based on *ACC*_*j*_. This procedure was repeated five times because the random separation of dataset #2 was conducted five times, and the mean threshold detection criterion for the five times repetition was calculated. Estimated label ***y*** was obtained against sensor output ***x*** in dataset #1, and the estimation score was calculated. Accuracy *ACC* was calculated by applying the mean threshold detection criterion to the estimation scores. Moreover, false alarm rates *F*_*j*_ and hit rates *H*_*j*_ were calculated, then ROC curves were plotted from (*F*_*j*_, *H*_*j*_). This procedure was repeated four times by changing dataset #1 for each participant, and the mean of accuracy *ACC* and the area under the curve *A*_g_ were calculated for four times validation.

#### Across-participant validation

Leave-one-out cross-validation was conducted against the data for all the participants. Figure 6B shows the procedure for the data preparation. The data for all the 40 trials from a single participant were used for the detection test (dataset #1). The remaining data were from 11 out of the 12 participants, and used for the model construction as dataset #2. Dataset #2 was divided into two groups: for the training (dataset #2-1) and for the determination of the detection criterion (dataset #2-2). For datasets #2-1 and #2-2, 26 trials from 13 of each type of trial, and 14 trials from 7 of each, were randomly selected from each participant, respectively. The entire procedure (training, determination of the criterion, detection test, and drawing of the ROC curves) was similar to the within-participant validation. The across-participant validation was independently conducted 12 times by changing dataset #1 along the participants.

## Results

### Within-participant validation

#### Detection performance

**Figure 7:**
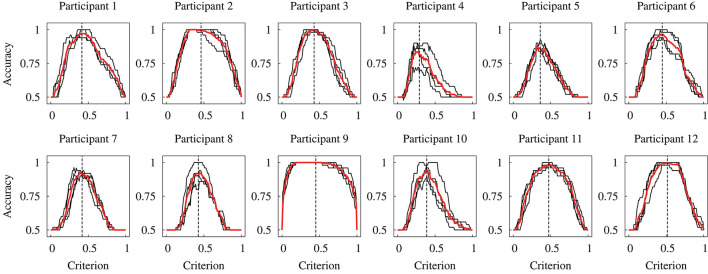
Relationship between the accuracy and detection criterion for each participant obtained in the within-participant validation. Black lines indicate each data for the 4-fold cross-validation, and red lines indicate the mean of four data. Black vertical dashed lines indicate the mean threshold criterion.

Figure 7 displays the relationship between the accuracy and detection criterion for each participant. This result was obtained against dataset #2; thus, it indicates the performance of the constructed model. The black and red solid lines show the results for four times validation and the mean, respectively. If a criterion has ultimate values (such as 0 or 1), the accuracy has the chance level of the detection (0.5). It can be seen that the relationship curve was different for the participants. Dashed vertical lines in Figure 7 represent the mean of the determined detection criteria. A higher accuracy is obtained at a criterion closer to the determined value.

**Figure 8:**
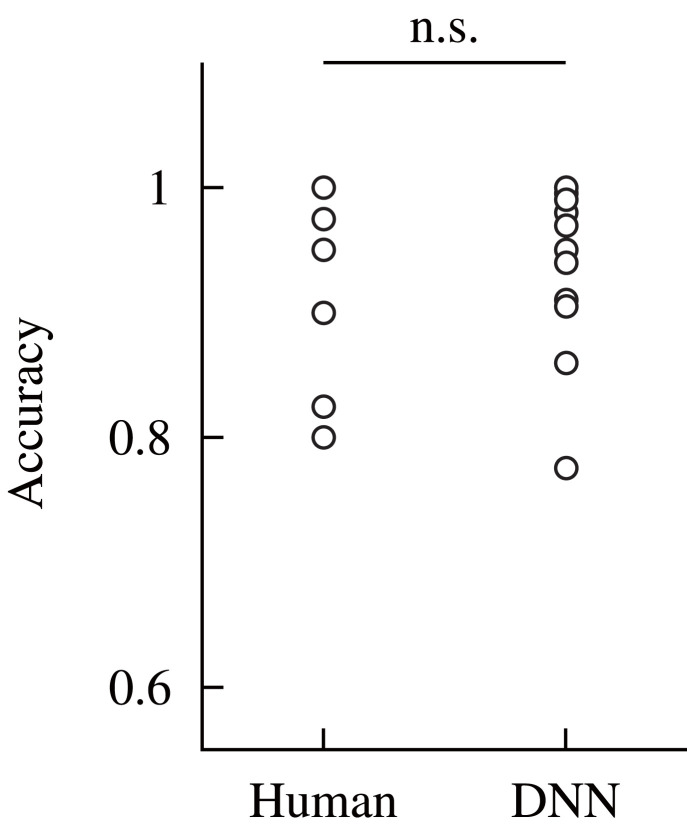
Comparison of the accuracy of the human participants and DNN model in the within-participant validation. The performance of the human participants was calculated based on the data from our previous study (Fukuda et al., 2018). Label n.s. indicates no significant difference at the 0.05 level with a Wilcoxon signed-rank test.

The accuracy was compared between the human participants and DNN model. Figure 8 shows the results of the comparison. The accuracy for the DNN model was obtained against dataset #1; thus, it indicates the detection performance of the model for the unknown dataset. The accuracy for the human participants was calculated based on the data obtained in our previous study (Fukuda et al., 2018). Statistical tests were conducted to compare the accuracy of the human participant and DNN model. Before the analysis, a Shapiro–Wilk test was conducted to confirm that the all-dependent parameters were normally distributed. Because the assumption of the normal distribution was violated, a Wilcoxon signed-rank test was conducted to compare the accuracy for the human participants and DNN model in the within-participant validation. The statistical test showed no significant difference in the accuracy of the human participants and DNN model [*W*_(12)_ = 23, *p* = 0.37]. This indicates that the median of the accuracy for the human participants and proposed DNN model were not different in the within-participant validation.

#### ROC curve

**Figure 9:**
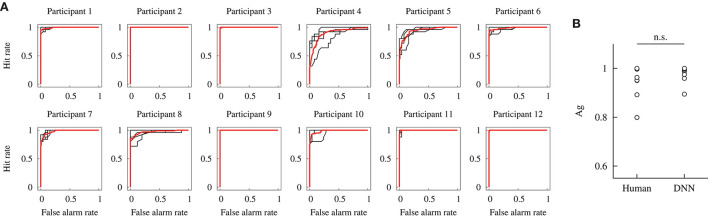
Potential detection performances of the DNN model constructed in the within-participant validation. **(A)** ROC curves. Black lines indicate each data for the 4-fold cross-validation, and red lines indicate the mean. **(B)** Comparisons of the area under the curve *A*_g_ of the human participant and DNN model. The performance of the human participants was obtained from our previous study (Fukuda et al., 2018). Label n.s. indicates no significant difference at the 0.05 level with a Wilcoxon signed-rank test.

Figure 9A depicts the ROC curves obtained against dataset #2 in the within-participant validation from all participants. Each curve is drawn based on the estimation by the DNN model, trained by the data from each participant. The black and red solid lines show the ROC curves for the 4-fold cross-validation and mean curves, respectively. If an ROC curve approaches the top left part, the curve indicates that the DNN model has a higher potential sensitivity. In particular, the ROC curves based on the data from participants 2, 9, and 12 indicate that the model achieved the ultimate potential sensitivity (*A*_g_ = 1) because the curves form a unit square.

The potential detection sensitivity *A*_g_ was compared for the human participants and DNN model. Figure 9B shows the comparison of *A*_g_. The values for the human participants were obtained in our previous study. A Wilcoxon signed-rank test showed no significant difference in the potential sensitivity between the human participants and DNN model [*W*_(12)_ = 33, *p* = 0.21]. It indicates that the median of the potential sensitivity for the human participants and proposed DNN model was not different in the within-participant validation.

### Across-participant validation

**Figure 10:**
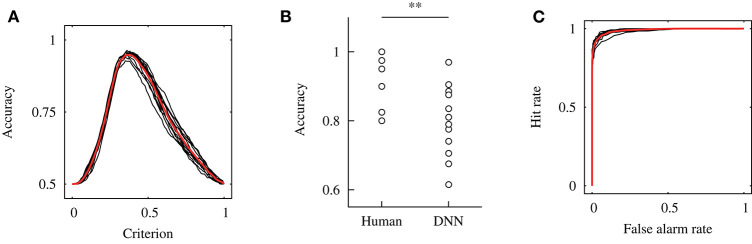
Results of the across-participant validation. **(A)** Relationship between the accuracy and detection criterion. Results of 12 independent validations shown by the black solid line, and their mean was shown by a red solid line. **(B)** Comparison of the accuracy for the human participants and DNN model. The accuracy for the human participants was calculated from our previous study (Fukuda et al., 2018). Here, ** indicates *p* < 0.01 with a Wilcoxon signed-rank test. **(C)** ROC curves.

Figure 10A exhibits the relationship between the accuracy and detection criterion in the across-participant validation. As the result was obtained against dataset #2, it indicates the performance of the constructed model. The black and red solid lines show each curve for 12 times validation and the mean curve, respectively. The mean and standard deviation of the determined criterion are represented as *c*_th_ = 0.38 ± 0.014, for which the detection performance of *ACC* = 0.95 ± 0.0093 was achieved.

Figure 10B shows a comparison of the accuracy for the human participants and DNN model in the across-participant validation. The accuracy of the model was calculated for dataset #1; thus, it indicates the performance of the model for the unknown dataset. A Wilcoxon signed-rank test showed a significant difference in the accuracy for the human participants and DNN model [*W*_(12)_ = 74, *p* = 0.006]. This result indicates that the median of the accuracy for the DNN model was significantly smaller than that for the human participants. Moreover, a one-sample Wilcoxon signed-rank test against 0.5 (the chance level of the detection) was conducted, and a significant difference was shown [*W*_(12)_ = 78, *p* = 0.002]. This indicates that the median of the accuracy for the DNN model was higher than the chance level.

Figure 10C shows the ROC curves obtained in the across-participant validation. This result was obtained against dataset #2. The black and red solid lines exhibit the data for each independent validation and mean of the results, respectively. The mean and standard deviation of the area under the curve were *A*_g_ = 0.99 ± 0.0037.

## Discussion

The experimental results of the within-participant validation showed no significant difference in the accuracies obtained for the human participants and proposed DNN model. This result indicates the possibility of replacing the detection by the participants based on visual feedback with the DNN model. Figure 7 shows that the relationship curve between the accuracy and various criteria were different for the participants. This difference might be due to a large variation in sensor output. In Figure 4 it can be seen that the sensor output that indicates the presence of a tumor was different for the participants, despite the same tumor being scanned. Moreover, we discovered in the previous study that the applied force and scanning speed varied between participants (Fukuda et al., 2018). Thus, the variation for the participants suggests a necessity for an appropriate determination of the detection criterion. We proposed usage of the relationship curve to determine the detection criterion. Here, Figure 9B shows that the potential sensitivities for the human participants and DNN model were similar in the within-participant validation. Even if the trained model has a high potential sensitivity, a high accuracy is not obtained until the detection criterion is not appropriate. This suggests that the proposed method to determine the criterion was appropriate. Moreover, Figure 7 shows that the relationship curve for participant 12 had more peaky shape in comparison with that for participant 9, whereas their potential detection sensitivities (ROC curves) were similar (Figure 9A). The more peaky shape suggests that the accuracy was more sensitive to changes in detection criterion. Thus, the relationship curve also reveals the robustness of the detection to a change in the detection criterion.

A comparison of Figures 8, **10B** shows that the accuracy for the DNN model in the within-participant validation tended to have a higher value than in the across-participant validation. Moreover, the accuracy for the model in the across-participant validation was significantly smaller than that for the human participants, whereas the mean potential detection sensitivity (area under the curve) of the constructed model was 0.99. This indicates that the model constructed in the across-participant validation achieved inefficient detection with unknown datasets. The inferior performance suggests that the use of data from the same participant is more effective in the training of the model. It could also be attributable to a variation in the appropriate detection criterion for the participants, as discussed in the above paragraph. In the across-participant validation, the criterion was determined from the data obtained from the other 11 participants. Thus, using the determined detection criterion might not be optimal for detection by the remaining participant, whose data was not used in the model construction. In contrast, the accuracy for the model was significantly higher than the chance level of the detection (0.5). Therefore, in practical applications it might be effective to use the data from the other users in the pre-training. This could contribute to more effective detection with low computational cost during the model update.

Next, we will discuss the future works toward clinical applications. Experiments with expert surgeons are necessary to investigate the applicable range and conditions of the proposed assistance algorithm at the next stage. Herein, the stiffness of the phantom used in the experiment lies within the stiffness range of the actual stomach wall and tumor (Fukuda et al., 2018); however, it did not have exactly the same anatomical structures and boundary conditions found in reality. In the experiment, it was shown that the novice participants could discriminate the phantom with/without the tumor after sufficient practice. Further, some participants achieved a complete detection performance. Thus, the experiment with expert surgeons in the current setup might not bring valuable discussion toward practical applications. In future work, we will investigate the applicable range and conditions of the algorithm through the sensor manipulation by expert surgeons in an *in-vivo* setup. In addition, it is important to consider the method of generating the tumor label for model training. In this paper, the label was calculated according to the measured positions of the phantom tumor and sensor. However, in surgical situations, it would be difficult to make the label in the same way, because the correct position of the tumor is not available during surgery. One solution is to record the time when the surgeon intraoperatively finds a tumor, according to feedback from the tactile sensor. If it is found in the postoperative examination that the position intraoperatively indicated by the surgeon is correct, then the label can be generated based on the recorded time and used for model training. Moreover, tumors vary in dimensions and stiffness, therefore it might be effective to input the preoperatively acquired features of the tumor to achieve more robust detection, regardless of tumor differences. In future work, we will develop a methodology for data acquisition considering surgical applications.

In our scenario, the user manipulates the tactile sensor and make a decision according to tactile feedback from the sensor, and the DNN model conducts the detection according to the sensor output in real-time. Thus, the proposed assistance algorithm is collaborative, and the surgeon should appropriately manipulate the sensor to scan the target tissue so that the effective detection by the model is achieved. Thus, it is necessary to optimize the tactile feedback of the sensor output to the surgeon, for more effective detection by both the surgeon and DNN-based assistant. Although our previous study showed that tactile feedback was only effective for a safer manipulation, effective tumor detection could be achieved by introducing the proposed assistance algorithm. We will also continue to improve the tactile display for sensory feedback, to improve decision making and sensor manipulation by the surgeon. Moreover, an important design factor for the data-driven assistant is the interaction between the surgeon and assistant. A possible method to realize this is by using an audio channel that is independent from the visual and tactile channels. Furthermore, the use of an audio channel would imitate the interaction between a surgeon and human assistant. Therefore, we will consider different approaches for achieving this interaction. Moreover, we will investigate the effect of this interaction on decision making and sensor manipulation by the surgeon.

## Conclusion

We proposed an assistance algorithm using a deep neural network for laparoscopic tumor detection. The algorithm uses the temporal output from a tactile sensor that is directly manipulated by a surgeon. Thus, safe and dexterous manipulation of the sensor is ensured by the surgeon, and the decision of the surgeon is assisted by the algorithm that performed the tumor detection simultaneously with and independently from the surgeon. This study was motivated by our previous psychophysical experiment, in which the participants had to detect a phantom of the stomach wall with/without a tumor based on visual feedback (a line graph based on the temporal sensor output). It was determined that providing visual feedback to an operator significantly enhanced the detection performance. However, using a visual channel for the sensory feedback was problematic because of the possibility of sensory overload, and of using valuable space in the operating room by the extra monitor. Thus, the proposed assistance algorithm in this study was intended to replace visual feedback.

A DNN model with three hidden layers was used to segment the sensor output, to identify whether the output included information on the tumor at each sampling. We proposed methods to input the temporal sensor output to the model considering real-time analysis, and to determine the detection criterion based on the relationship between accuracy and the various detection criteria. Moreover, signal detection theory was employed to assess the potential detection sensitivity of the model. Subsequently, two types of validation (within-participant and across-participant validation) were conducted to assess the effectiveness of the proposed model. Further, the detection performances were compared for the DNN model and human participants, which were obtained in our previous study. The results of the within-participant validation showed no significant differences in the accuracy and potential sensitivity for the human participants and DNN model. Thus, the possibility of replacing the detection by a user with a DNN-based algorithm was shown. Although the results in the across-participant validation showed that the accuracy of the DNN model was significantly less than that of the human participants, the performance was significantly higher than the chance level of the detection. Thus, this suggests that the data obtained from other users might be used in pre-learning of the model.

In future work, we will investigate the applicable range and conditions of the proposed algorithm. Moreover, we will consider a method for data collection in surgical situations. We will also develop an effective method to interact with the DNN-based assistant, and investigate the effects of the assistant on decision and sensor manipulation by the surgeon.

## Data availability statement

The datasets analyzed for this study can be found in the figshare at https://figshare.com/s/a70b08558fced7cca9c9.

## Author contributions

TF and YT contributed to the conception of the study. TF organized the dataset, developed the algorithm, and performed the validation. TF and YT contributed to the analysis, the interpretation of the results, and wrote the first draft of the manuscript. MF and AS supervised the study. All authors contributed to manuscript revision, read and approved the submitted version.

### Conflict of interest statement

The authors declare that the research was conducted in the absence of any commercial or financial relationships that could be construed as a potential conflict of interest.
